# Postoperative pain control after free gingival graft: a systematic review and meta-analysis of RCTS

**DOI:** 10.1590/0103-644020256484

**Published:** 2025-12-01

**Authors:** Julio Rebollal, Dennis Malta Guimarães, Erika Romanini, Vittorio Moraschini, José Mauro Granjeiro

**Affiliations:** 1 Latin American Institute of Dental Research, ILAPEO, Curitiba, Paraná Brasil; 2 School of Dentistry, Federal Fluminense University, Niterói, Rio de JaneiroBrasil

**Keywords:** Free gingival graft, Palatal wound healing, Wound epithelization, Palate

## Abstract

This systematic review and meta-analysis evaluated the efficacy of interventions for managing postoperative pain following a free gingival graft (FGG) harvested from the palate. A comprehensive search was conducted in eight major databases, without language restrictions, following PRISMA guidelines. Randomized clinical trials (RCTs) involving adults undergoing FGG and assessing postoperative pain were included. Pain was evaluated using the Visual Analog Scale (VAS). Risk of bias was assessed using the Cochrane RoB 2 tool. The protocol was registered on INPLASY (ID INPLASY2022120077). Among 48 studies, 16 had a low risk of bias, 27 presented some concerns, and 5 showed a high risk, mainly related to blinding and allocation concealment. The analysis identified Leukocyte-Platelet Rich Fibrin (L-PRF) + Sutures as the most effective intervention for pain reduction on Day 1 (-6.80; 95% CI [-7.68, -5.92]) compared to sutures and surgical cement. Additionally, Gelatin Sponge + 0.2% Hyaluronic Acid + Sutures significantly reduced pain (-4.80; 95% CI [-6.03, -3.57]) compared to Gelatin Sponge + Sutures. Heterogeneity among studies was substantial (I² = 78-92%), indicating variability in study designs and outcomes, affecting generalizability. Funnel plot analysis suggested a moderate risk of publication bias, potentially overestimating treatment effects. L-PRF and 0.2% HA gel were the most effective interventions for postoperative pain reduction. Palatal protection plates emerged as a viable alternative in resource-limited settings due to their ease of fabrication and affordability. However, substantial heterogeneity and moderate risk of publication bias highlight the need for further high-quality trials.

**Figure:**
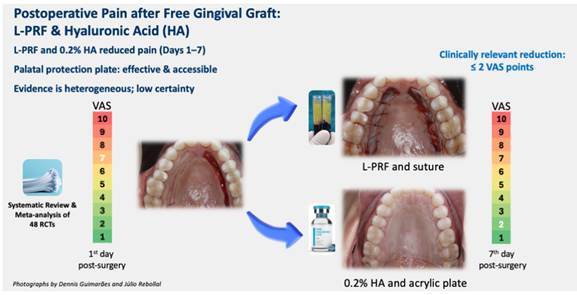

## Introduction

Autogenous gingival grafts are widely used in periodontal plastic surgery to address various clinical needs. These grafts are primarily indicated for gingival recession coverage, increasing the width and thickness of keratinized gingiva, managing recessions around implants, correcting deficiencies in keratinized tissue and volume around implants, and generally enhancing oral mucosal tissues. Connective tissue grafts are typically harvested from donor sites such as edentulous ridges, the maxillary tuberosity, and most commonly, the palate. The palate is favored due to the ample tissue availability and its histological similarity to keratinized gingiva attached to the alveolar crest ^1^.

Various graft harvesting techniques have been documented in the literature, with the free gingival graft (FGG) and the subepithelial connective tissue graft (SCTG), along with their variations, being the most commonly employed methods ^2^ ^,^ ^3^ ^,^ ^4^ ^,^ ^5^. The FGG technique, in particular, is popular due to its relative ease of removal, the greater availability of tissue, and its applicability even in areas with thinner palatal mucosa. Despite these advantages, a significant limitation of the FGG method is the healing process at the donor site, which occurs by secondary intention and typically requires a period of two to four weeks. This prolonged healing period often results in considerable discomfort and pain for patients. As a result, a variety of interventions have been proposed to alleviate postoperative pain.

These interventions range from sutures, eugenol-free surgical cements, hemostatic periodontal dressings, absorbable collagen and gelatin sponges, protective stents, photobiomodulation, leukocyte- and platelet-rich fibrin (L-PRF), hyaluronic acid gels, ozone therapy, electrotherapy, hemostatic plant extracts, to tissue adhesives. However, the comparative efficacy of these diverse options remains inconclusive, and there is no consensus in the literature on the most effective strategies for minimizing pain after FGG harvesting from the palate ^6^.

Given this notable gap in the current understanding, this systematic review and meta-analysis aim to assess and compare the efficacy of different interventions in pain management following the removal of free gingival grafts from the palate. This study seeks to provide a comprehensive, evidence-based analysis to inform clinical practice, ultimately aiming to enhance patient outcomes in periodontal and dental surgeries.

## Materials and methods

### Protocol and Registration

This systematic review was conducted in accordance with the "Cochrane Handbook for Systematic Reviews of Interventions". It adhered to the "Preferred Reporting Items for Systematic Reviews and Meta-Analyses" (PRISMA) checklist to ensure transparency and comprehensiveness in reporting. The protocol was registered with the "International Platform of Registered Systematic Review and Meta-Analysis Protocols" (INPLASY) on December 19, 2022, under registration number INPLASY2022120077. In accordance with the registered protocol (INPLASY2022120077), no deviations or amendments were made during the conduct of this systematic review and meta-analysis.

### Focused Question

The primary question guiding this systematic review is: "What is the most effective procedure for pain control following the removal of a free gingival graft from the palate donor area in humans?" This question aims to provide clear and objective answers that are essential for improving clinical procedures in periodontics. To structure this inquiry systematically, the PICOS model was adopted, encompassing Population (adult individuals who underwent free gingival grafts with palatal donor sites), Intervention (application of auxiliary therapies to reduce pain in palatal wounds after removal of free gingival grafts from the palate), Comparison (internal study comparator), Outcome (postoperative pain), and Study Design (randomized clinical studies). This model guides both the formulation of the research question and the strategy for searching and analyzing the included studies.

### Eligibility Criteria

To ensure relevance and quality, the study applied strict eligibility criteria, focusing on study design, selection parameters, and research approach. The review included only randomized clinical trials (RCTs) with adult participants over 18 years, without language restrictions. Studies are needed to assess postoperative pain management after free gingival graft removal from the palatal donor area.

The review excluded studies that did not meet these criteria. In vitro and in vivo animal studies, literature reviews, systematic reviews, non-randomized clinical studies, and observational studies, including cohorts, case-controls, and case series, did not qualify. Additionally, narrative reports and studies with incomplete or missing data did not fit the selection criteria.

### Databases Consulted, Search Strategies, and Article Selection Process

**Figure 1:**
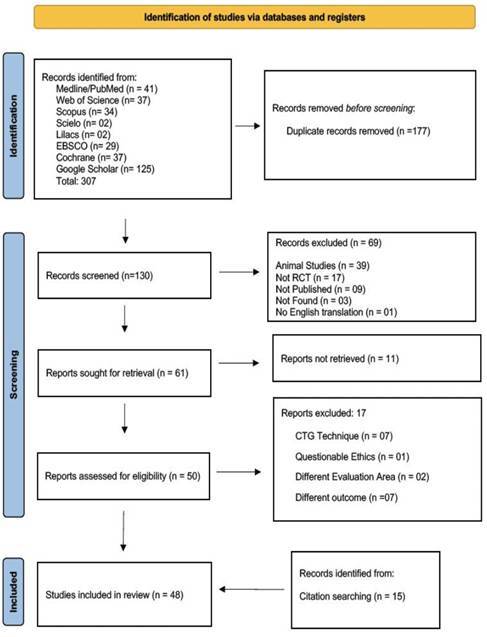
PRISMA Flowchart of Study Selection Process.

To ensure a comprehensive review, an extensive search was conducted across multiple recognized databases, including the National Library of Medicine (PubMed/Medline), Scientific Electronic Library Online (SCIELO), LILACS, EBSCO, Web of Science, Scopus, and Cochrane. For grey literature, searches were also performed in Google Scholar. The initial search was performed on October 20, 2023, and was updated on September 15, 2024. The search strategy utilized a combination of Medical Subject Headings (MeSH) terms and synonyms relevant to the PICO-modeled research question, refined with Boolean operators (AND, OR). For PubMed, the search query used was ((("free gingival graft"[All Fields]) OR ("connective tissue graft"[All Fields])) and (("palatal wound healing"[All Fields]) OR ("wound epithelization"[All Fields]))). For other databases and Google Scholar, the query used was ((("free gingival graft") OR ("connective tissue graft")) and (("palatal wound healing") OR ("wound epithelization"))). This approach ensured both specificity and inclusivity in capturing relevant studies from a wide range of sources, including both published studies and grey literature. Details of the final search strategy, including the number of works found in each database, are presented in Figure 1.

Following the initial search, duplicates were removed using Mendeley Reference Manager (Mendeley®, Elsevier, London, UK). Two independent evaluators (JR and DG) conducted the initial screening of titles and abstracts using the Mendeley platform and the RAYYAN application. Any disagreements during this phase were resolved by consensus to ensure a rigorous and unbiased selection.

Subsequently, the same evaluators proceeded to read the full articles. Considering the previously defined eligibility criteria, they selected potentially eligible studies, deciding on their final inclusion in the data extraction, as per the 2020 “Prisma Flow.” Any conflicts arising during this phase were resolved by consensus, ensuring a balanced and objective assessment.

### Data extraction

Data extraction was performed independently by two reviewers (JR and DG) using a standardized data extraction form. In cases where discrepancies arose between the reviewers, a third expert (JMG) was consulted to achieve consensus. This approach ensured the accuracy and reliability of the data extraction process.

From each included study, a comprehensive set of data items was extracted to ensure a robust analysis. The extracted data included: ^1^ study characteristics (e.g., authors, year of publication, country); ^2^ sample size and design details (e.g., split-mouth, parallel); ^3^ participant characteristics (e.g., gender, mean age, age range); ^4^ details of control and intervention groups (e.g., type of intervention, specific agents used like L-PRF, hyaluronic acid, etc.); ^5^ follow-up periods for pain; ^6^ methods used pain assessments (e.g., Visual Analog Scale (VAS), ^7^ results for primary outcomes (e.g., pain scores); and ^8^ any additional assessments.

### Outcome Measures

The primary outcome for this systematic review was postoperative pain at the donor site after free gingival graft (FGG) removal. These outcome was chosen because they are the most directly relevant to clinical decision-making in periodontics and peri-implant surgery. Effective management of postoperative pain is crucial for patient comfort and compliance. Pain was assessed using the Visual Analog Scale (VAS, from 1 to 10), a widely used tool that allows for quantification of subjective pain experience. The synthesis of results involved subgroup meta-analyses for different time points (e.g., days 1, 3, and 7 for pain assessment) to account for changes over time, and data were pooled using random-effects models to manage heterogeneity among studies.

### Synthesis Method

A meta-analysis was performed using a random-effects model due to the substantial heterogeneity observed among the included studies, as indicated by an I² value of 87.1%. The random-effects model was chosen because it accounts for variability both within and between studies, making it more appropriate when the included studies differ in terms of interventions, populations, or outcome assessment methods. This approach provides a more conservative estimate of the overall effect size and acknowledges that the actual effect may vary from study to study. To further explore the impact of heterogeneity on the results, subgroup analyses were conducted based on the type of comparator and follow-up period, and sensitivity analyses were performed by excluding studies with a high risk of bias. These additional analyses helped to assess the robustness of the findings and understand how variations in study characteristics might influence the pooled effect estimates.

### Assessment of Bias Risk

The quality of the included studies was assessed using the Cochrane Risk of Bias 2 (RoB 2) tool, as outlined in the Cochrane Collaboration's Handbook for systematic reviews of interventions. The risk of bias for each included study was assessed using the Cochrane Risk of Bias tool 2 (RoB 2), which evaluates five domains: ^1^ bias arising from the randomization process, ^2^ bias due to deviations from intended interventions, ^3^ bias due to missing outcome data, ^4^ bias in the measurement of the outcome, and ^5^ bias in the selection of the reported result. Two independent reviewers (JR and DG) assessed the risk of bias for each study. Discrepancies were resolved through consensus with a third expert (JMG). Each domain was rated as 'low risk', 'some concerns', or 'high risk'. Studies rated as 'some concerns' indicate potential biases that could slightly affect the confidence in the results, whereas studies rated as 'high risk' suggest substantial potential for bias, which could significantly impact the reliability of the findings.

### Certainty of Evidence Assessment

The certainty of evidence for pain outcomes (measured by Visual Analog Scale) on postoperative days 1, 3, and 7 was assessed using the GRADE (Grading of Recommendations, Assessment, Development and Evaluations) approach. The assessment considered five domains: risk of bias, inconsistency, indirectness, imprecision, and publication bias. All included studies were randomized controlled trials (RCTs), and the certainty began as high for each outcome. Two reviewers independently evaluated each domain, and disagreements were resolved by consensus. The assessment focused only on studies included in the meta-analyses, as per GRADE guidance.

To assess safety, each included study was reviewed for any mention of adverse events or complications by screening the results, discussion, and limitations sections.

### Statistical analysis

Continuous variables were analyzed in a meta-analysis using Review Manager (version 5.2.8, Copenhagen, Denmark, 2014). The effects of the interventions were estimated using the mean difference (MD) with a 95% confidence interval (CI). A generic inverse variance method was applied to calculate the pooled effect sizes. Heterogeneity among the studies was assessed using the Chi² test and quantified by the I² statistic. We defined heterogeneity levels as follows: low heterogeneity for I² values ≤ 25%, moderate heterogeneity for values > 25% and ≤ 50%, and high heterogeneity for values > 50%. Given the variability in the available evidence, such as differences in study populations, follow-up durations, and clinical settings, a random-effects model was employed for the meta-analyses. This approach accounts for potential variations across studies, providing more generalized and robust estimates. The threshold for statistical significance in the meta-analysis was set at P <0.05.

### Results

### Study Characteristics

The initial search across eight major databases and Google Scholar yielded a total of 307 references up to October 20, 2024. After removing duplicates, 130 unique articles remained. Using the RAYYAN tool, 69 articles were excluded by consensus for not meeting the eligibility criteria. Of the 61 titles selected for retrieval, 11 were unobtainable as they had not yet been published. Consequently, 50 articles were subjected to full-text review, and an additional 15 articles were manually included after a bibliographic reference analysis of the selected articles. Following a detailed assessment, 48 articles were consolidated for qualitative synthesis. In total, 259 irrelevant titles and abstracts were discarded during the screening process. The systematic selection process is summarized in the PRISMA Flowchart (Figure 1).

Primary data of interest, including the general characteristics of the studies (authors, year of publication, country, and study design), sample size, specificities of the study groups (interventions in control and test groups), population characteristics (mean age, gender, country of origin), follow-up duration for each outcome (pain and healing), and the assessment methods used, were extracted and systematized in Supplementary Table 1. A total of 1711 participants underwent de-epithelialized free gingival grafts, comprising 491 men and 945 women, resulting in a total of 1782 samples. Notably, seven articles did not report the gender of participants ^7^ ^,^ ^8^ ^,^ ^9^ ^,^ ^10^ ^,^ ^11^ ^,^ ^12^ ^,^ ^13^. The participants' ages ranged from 18 to 78 years, with five studies not reporting age ^7^ ^,^ ^8^ ^,^ ^12^ ^,^ ^14^ ^,^ ^15^.

The sample sizes of the studies varied considerably, ranging from 10 to 125 participants. Five studies employed the split-mouth technique, where each patient acts as their own control ^8^ ^,^ ^10^ ^,^ ^16^ ^,^ ^17^ ^,^ ^18^. For studies that assessed pain, five utilized the visual analog scale (VAS) ranging from 0 to 100 mm ^13^ ^,^ ^17^ ^,^ ^19^ ^,^ ^20^ ^,^ ^21^, one used the VAS scale from 0 to 5 ^22^, one employed a numerical pain scale (NPS) ^23^ and one used a numerical rate scale (NMRS) with 0.5 increments ^24^. The remaining studies used a VAS scale from 0 to 10.

The analysis of pain management strategies revealed various approaches among the control groups. Eight articles used only sutures as the control ^14^ ^,^ ^16^ ^,^ ^25^ ^,^ ^26^ ^,^ ^27^ ^,^ ^28^ ^,^ ^29^ ^,^ ^30^, while eight studies had no specific intervention in the control group ^9^ ^,^ ^10^ ^,^ ^31^ ^,^ ^32^ ^,^ ^33^ ^,^ ^34^ ^,^ ^35^ ^,^ ^36^. Eight studies utilized gelatin sponges with sutures as the control ^20^ ^,^ ^24^ ^,^ ^37^ ^,^ ^38^ ^,^ ^39^ ^,^ ^40^ ^,^ ^41^ ^,^ ^42^, and five randomized controlled trials (RCTs) employed surgical cement for the control group ^19^ ^,^ ^26^ ^,^ ^43^ ^,^ ^44^. The palatal protective plate was used in seven articles ^8^ ^,^ ^12^ ^,^ ^18^ ^,^ ^45^ ^,^ ^46^ ^,^ ^47^ ^,^ ^48^, and five studies selected collagen sponges combined with sutures ^22^ ^,^ ^26^ ^,^ ^42^ ^,^ ^49^ ^,^ ^50^.

Less common control interventions included chitosan and protective plates ^7^ ^,^ ^51^, aluminum foil combined with surgical cement ^21^, surgical cement alongside a Hawley-type protective plate ^52^, a combination of collagen sponge, absorbable sutures, surgical cement, and standard sutures ^53^, petroleum jelly as a placebo ^13^, Revitalized Oxidized Cellulose ^17^, and sutures with surgical cement ^54^.

### Risk of Bias Assessment

**Figure 2:**
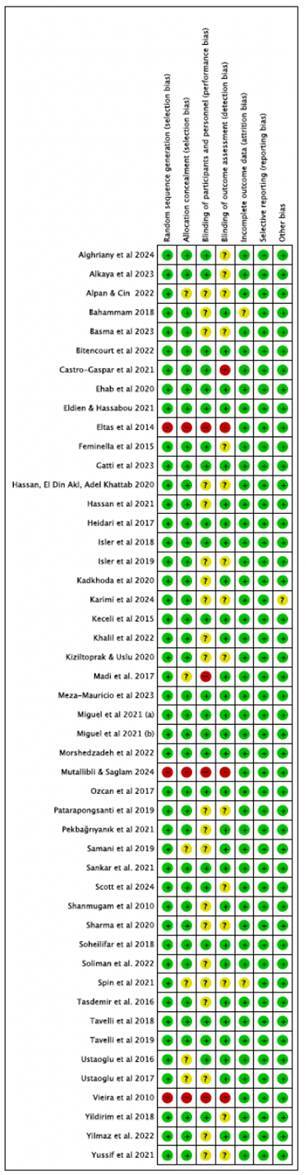
Bias Risk Summary: Authors' judgments on each risk of bias domain for each included study.

Assessing bias played a crucial role in ensuring the reliability and validity of the results (Figure 2). Among the 48 articles analyzed, 16 contained only items classified as having a low risk of bias. Sixteen articles were classified as having only items at low risk of bias ^7^ ^,^ ^8^ ^,^ ^9^ ^,^ ^16^ ^,^ ^26^ ^,^ ^27^ ^,^ ^28^ ^,^ ^29^ ^,^ ^34^ ^,^ ^35^ ^,^ ^37^ ^,^ ^41^ ^,^ ^50^ ^,^ ^51^ ^,^ ^55^ ^,^ ^56^. Fourteen articles had one domain flagged with some concerns ^11^ ^,^ ^12^ ^,^ ^15^ ^,^ ^20^ ^,^ ^23^ ^,^ ^24^ ^,^ ^30^ ^,^ ^38^ ^,^ ^42^ ^,^ ^43^ ^,^ ^44^ ^,^ ^47^ ^,^ ^53^ ^,^ ^57^, while ten had two domains with similar concerns ^10^ ^,^ ^17^ ^,^ ^21^ ^,^ ^22^ ^,^ ^31^ ^,^ ^40^ ^,^ ^45^ ^,^ ^46^ ^,^ ^49^ ^,^ ^54^. Two articles presented issues in three domains ^18^ ^,^ ^33^, and one article showed concerns in four domains ^52^. Regarding high-risk classifications, two articles contained one domain rated as high risk of bias ^13^ ^,^ ^14^, whereas three articles exhibited high risk in four domains ^19^ ^,^ ^36^ ^,^ ^48^.

**Figure 3:**
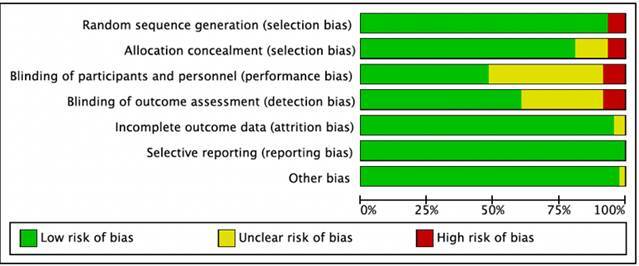
Bias Risk Chart: Authors' judgments on each risk of bias domain presented as percentages across all included studies.

A detailed analysis of the bias domains classified as "some concerns" or "high risk" provided further insights into the methodological quality of the studies (Figure 3). For domains rated as some concerns, 39.6% were related to the blinding of participants and study personnel, 28.3% of cases were due to issues with the blinding of outcome assessment, 11.3% were associated with allocation concealment, 3.7% were pertinent to incomplete data, and 1.8% were linked to other bias. The domains rated as high risk were related to several critical areas, including randomization process (5.6%), allocation concealment (5.6%), blinding of participants and study personnel (7.5%), and blinding of outcome assessment (7.5%).

### Certainty of Evidence and Effect Estimates

The certainty of evidence for pain outcomes on postoperative days 1, 3, and 7 was evaluated using the GRADE approach (Table 1). Across all timepoints, the evidence was downgraded due to serious concerns regarding risk of bias (e.g., absence of blinding and incomplete reporting), inconsistency (notable heterogeneity among studies), and imprecision (wide confidence intervals and small sample sizes). No concerns were identified regarding indirectness. However, concerns regarding publication bias were noted based on asymmetry in the funnel plot analysis, suggesting potential overestimation of effect sizes.

The pooled analysis demonstrated a statistically significant reduction in postoperative pain in favor of the experimental interventions compared to the control. On day 1, the mean difference was -2.32 points on the visual analog scale (95% CI: -3.56 to -1.07); on day 3, -2.02 (95% CI: -2.86 to -1.18); and on day 7, -1.77 (95% CI: -2.44 to -1.09). Although these findings indicate consistent effects, the overall certainty of the evidence for each estimate was rated as very low.

**Table 1:** GRADE Summary Table - Pain Outcome (Days 1, 3, 7)

Day	Studies	Experimental design	Risk of Bias	Inconsistency	Indirect Evidence	Inaccuracy	Other considerations	Participants (Exp)	Participants (Ctrl)	Effect (95% CI)
Day 1	11	RCTs	Serious	Serious	Not serious	Serious	Potential publication bias	217	199	MD -2.32 [-3.56, -1.07]
Day 3	10	RCTs	Serious	Serious	Not serious	Serious	Potential publication bias	215	198	MD -2.02 [-2.86, -1.18]
Day 7	15	RCTs	Serious	Serious	Not serious	Serious	Potential publication bias	285	255	MD -1.77 [-2.44, -1.09]

Exp - experimental group; Ctrl - control group; CI - confidence interval

### Pain Analysis

In our systematic review of 48 articles, two studies lacked both mean and standard deviation data for pain outcomes ^13^ ^,^ ^46^, and 1 study presented results in percentages ^52^. Nine articles included graphical data, but the data could not be precisely extracted from these graphs ^16^ ^,^ ^26^ ^,^ ^27^ ^,^ ^28^ ^,^ ^34^ ^,^ ^36^ ^,^ ^49^ ^,^ ^54^ ^,^ ^56^. Six studies reported mean pain values without standard deviations ^9^ ^,^ ^13^ ^,^ ^17^ ^,^ ^32^ ^,^ ^51^ ^,^ ^55^, and five studies used median values to present pain data ^23^ ^,^ ^33^ ^,^ ^41^ ^,^ ^43^ ^,^ ^53^. One study provided mean and standard deviation values only for the 30th postoperative day ^45^.

Efforts were made to acquire complete data from studies with unclear graphical data ^9^ ^,^ ^16^ ^,^ ^17^ ^,^ ^26^ ^,^ ^27^ ^,^ ^28^ ^,^ ^34^ ^,^ ^49^ ^,^ ^54^ ^,^ ^55^ ^,^ ^56^. The corresponding authors were contacted via email on August 20, 2023. Only two authors responded; one provided complete data for two articles ^27^ ^,^ ^28^, while the other indicated that the requested data were unavailable, resulting in that study being excluded from the meta-analysis ^55^. After four weeks, articles from non-responding authors were excluded from the quantitative analysis ^9^ ^,^ ^16^ ^,^ ^17^ ^,^ ^26^ ^,^ ^34^ ^,^ ^49^ ^,^ ^54^ ^,^ ^56^.

Among the 23 articles providing complete postoperative pain data (mean and standard deviation) evaluated up to the 14th day, two studies used a Visual Analog Scale (VAS) of 0 to 100 mm ^14^ ^,^ ^21^, 1 study used a VAS scale of 0 to 5 ^22^, and 1 RCT used a Numerical Rate Scale (NMRS) of 0 to 10 but with intervals of 0.5 ^24^. These studies were excluded from the meta-analysis to maintain consistency in data presentation and comparison.

**Figure 4:**
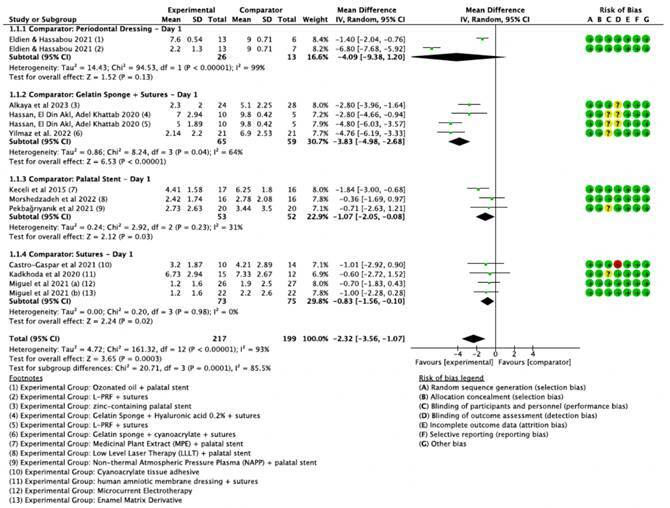
Forest plot illustrating the mean differences in postoperative pain on Day 1 between various experimental groups and their respective comparator groups.

**Figure 5:**
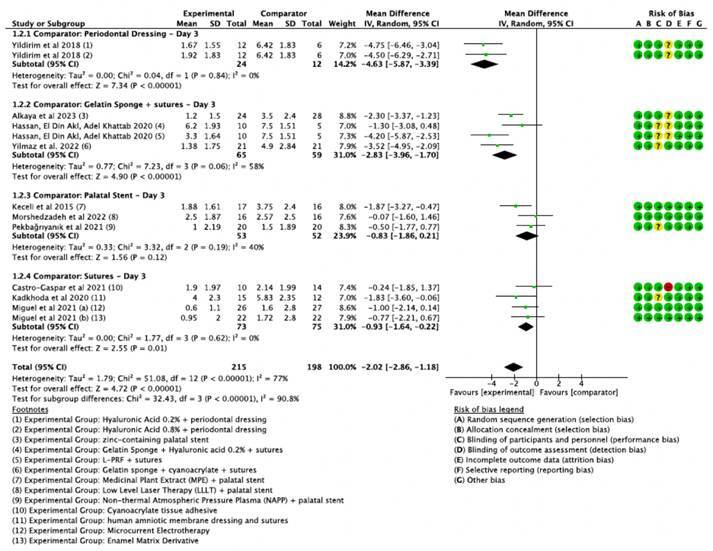
Forest plot illustrating the mean differences in postoperative pain on Day 3 between various experimental groups and their respective comparator groups.

**Figure 6:**
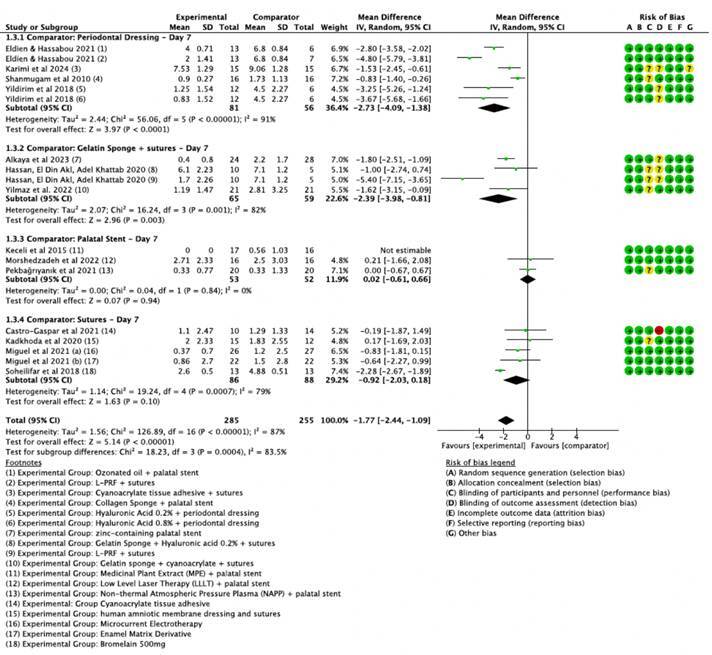
Forest plot illustrating the mean differences in postoperative pain on Day 7 between various experimental groups and their respective comparator groups.

Of the articles selected for the meta-analysis, interventions were analyzed across different studies: Leukocyte-Platelet Rich Fibrin (L-PRF) in four articles ^7^ ^,^ ^20^ ^,^ ^31^ ^,^ ^43^, Low-Level Laser Therapy (LLLT) in two articles Ozone Therapy in two randomized controlled trials ^7^ ^,^ ^34^, and 0.2% hyaluronic acid in two studies ^43^ ^,^ ^44^. These studies demonstrated significant heterogeneity. To address this, studies were stratified into subgroups based on control group types and follow-up times (1st, 3rd, and 7th postoperative days), as presented in Figures 4, 5, and 6 .

On the 1st postoperative day (Figure 4), pain assessment revealed that the L-PRF group ^7^, particularly when compared to sutures and surgical cement (-6.80, CI95% [-7.68, -5.92]), was the most effective in reducing pain. However, when comparisons involved gelatin sponge + sutures as the control, there were no significant differences between L-PRF, hyaluronic acid 0.2%, and collagen sponge + cyanoacrylate + sutures, although all treatments were more effective than the control ^38^ ^,^ ^40^ and presented a combined effect of -3.83 (CI 95% [-4.98, -2.68]). In subgroups where no specific intervention was used as the control, L-PRF did not demonstrate superior effectiveness. Interestingly, pain levels with palatal protection plates as a control (subgroup 1.1.3 in Figure 4) were as low as those of active interventions like LLLT and Non-Ablative Plasma Pen (NAPP), indicating that the plate itself acts as an active comparator.

Overall, on the 3rd postoperative day (Figure 5), when sutures combined with surgical dressing were used as the comparator, the most significant reduction in pain was observed in intervention groups using hyaluronic acid at concentrations of 0.2% and 0.8% (-4.63, CI 95% [-5.87, -3.39]), with no statistically significant difference noted between these concentrations. However, compared to gelatin sponge plus sutures, hyaluronic acid 0.2% did not show superior outcomes (-1.30, CI 95% [-3.08, 0.48]). In this subgroup, L-PRF combined with sutures, zinc-containing palatal stent, and collagen sponge + cyanoacrylate + sutures were notably more effective on the third day postoperatively than the control group of gelatin sponge + sutures (subgroup 1.2.2).

On the 7th postoperative day, L-PRF also appeared more effective compared to surgical cement plus sutures, though it was not statistically different from 0.2% and 0.8% hyaluronic acid concentrations (Figure 6, subgroup 1.3.1), with an overall effect of -2.73 (CI 95% [-4.09, -1.38]). However, L-PRF showed statistically significant superiority over hyaluronic acid when the comparator was gelatin sponge plus sutures (subgroup 1.3.2) with a -5.40 reduction in pain (CI 95% [-7.15, -3.65]).

At all evaluated time points, pain levels in the palatal protection plate subgroup (subgroup 1.1.3) remained comparable to active interventions, including LLLT, Non-Ablative Plasma Pen (NAPP), and the Medicinal Plant Extract (MPE), with no significant differences between the palatal plate group and other treatments.

**Figure 7:**
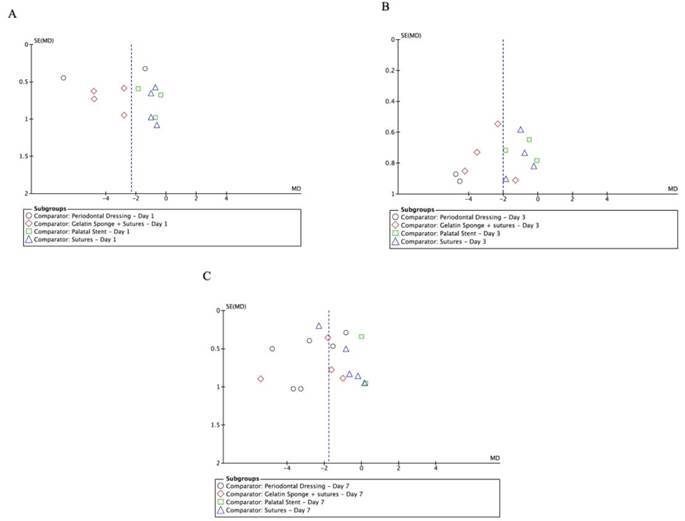
Funnel Plots for postoperative pain analysis on Days 1 (A), 3 (B), and 7 (C). The central line in each plot indicates the average change in reported pain: Day 1 (MD = -2), Day 3 (reduction relative to various interventions), and Day 7 (MD close to zero). Points represent individual studies with their mean differences in pain and standard errors. Distinct shapes in each plot correspond to the different comparators used in the subgroups.

The funnel plot analysis indicated a degree of heterogeneity and possible publication bias in studies assessing postoperative pain on day one. The asymmetrical distribution of study points (Figure 7A) suggests varying external influences on the data. On the 3rd day, the funnel plot displayed moderate variation in pain reduction across studies (Figure 7B), with most studies reporting some level of pain relief. By the 7th day, the funnel plot showed that the overall effect of interventions on pain was minimal, with study points scattered around the no-effect line (Figure 7C), underscoring the complexity of managing long-term postoperative pain.

## Discussion

Periodontal and peri-implant surgical techniques have significantly evolved since their initial descriptions, with expanded indications and refined graft harvesting approaches^58^.

A critical aspect of these surgeries is managing postoperative morbidity, particularly pain and discomfort, which can discourage patients from undergoing necessary treatments. Alternatives such as allografts, xenografts, and synthetic soft tissue substitutes, including collagen matrices, have been explored to minimize morbidity. However, these alternatives generally provide less volume gain compared to the traditional FGG^59^.

Our systematic review shed light on the variability and efficacy of techniques used to reduce postoperative pain. The focus was on the de-epithelialization technique ^1^, which, although potentially more invasive and painful ^2^ ^,^ ^5^, did not show significant differences in terms of analgesic consumption, postoperative discomfort, and bleeding when compared to the SCTG technique ^1^.

The review revealed substantial heterogeneity among the studies regarding methods, assessment periods, control groups, populations, and graft dimensions. The Visual Analog Scale (VAS) was predominantly used for pain assessment, highlighting its sensitivity to small changes in pain intensity, despite some limitations, such as variability in patient interpretation and reporting ^60^. Blinding of outcome assessment and the blinding of participants and personnel were identified as the most unclear domains in the evaluated RCT, which could influence subjective outcomes like postoperative pain.

Our findings corroborate the efficacy of L-PRF in reducing postoperative pain. The beneficial effects of L-PRF, attributed to its growth factors and cellular content, are crucial for enhancing surgical wound repair ^61^. The review also suggested that the use of surgical cement as a comparator might have acted as a local irritant, thereby influencing outcomes that favored L-PRF ^7^. However, Gatti et al. ^37^ found no significant advantage of L-PRF over Oxidized Regenerated Cellulose in reducing pain, suggesting that both materials could be effective options for managing postoperative discomfort.

Interestingly, when comparing L-PRF with a no-intervention control group, no significant differences in pain reduction were observed ^31^. Hyaluronic acid at 0.2% was found to be comparable to L-PRF on days 1 and 3, particularly when gelatin sponges with sutures were used as comparators ^38^ ^,^ ^40^ ^).^

This finding suggests its potential as an alternative treatment for managing pain. However, the need for specialized equipment for L-PRF preparation can be a financial barrier, positioning alternatives such as collagen sponges or 0.2% hyaluronic acid (HA) gel as more accessible and practical options for some clinical settings.

The consistent pain reduction observed in the palatal protection plate subgroup across all evaluated timepoints suggests that the plate itself may contribute to postoperative comfort by minimizing mechanical trauma and irritation at the surgical site. The observed reductions in pain exceeded 2 points on the VAS scale, which surpasses the minimal clinically important difference (MCID) for postoperative pain, indicating a meaningful benefit from the patient's perspective. These findings highlight the potential clinical relevance of palatal plates as a simple, non-invasive, and cost-effective alternative for pain management in free gingival graft procedures. Further studies are warranted to understand their role in postoperative recovery better and compare their efficacy to more advanced interventions.

In this systematic review, the presence of studies with 'some concerns' and 'high risk' of bias in key domains could affect the overall confidence in the findings. Studies classified as 'high risk' were carefully considered when interpreting the meta-analytic results, and sensitivity analyses were conducted to assess the robustness of the findings when these studies were excluded. As a result, while L-PRF and hyaluronic acid gel showed significant effectiveness in reducing postoperative pain, the strength of this evidence is moderated by the risk of bias identified in certain studies. The use of different control materials (e.g., sutures, surgical cement) likely influenced pain perception. Some materials, such as surgical cement, may act as irritants, possibly exaggerating the observed benefits of tested interventions. This clinical variability was considered in subgroup analyses and contributed to the GRADE downgrading.

The funnel plot analysis indicated potential publication bias, as suggested by the asymmetrical distribution of studies, particularly for the outcomes related to postoperative pain on Day 1. This asymmetry might suggest that smaller studies with negative or non-significant findings are underrepresented in the literature. Consequently, the pooled effect size estimates for pain reduction might be slightly overestimated. To further explore this, sensitivity analyses were performed, which included recalculating the overall effect sizes by excluding smaller studies and those with a high risk of bias. These analyses showed that while the overall direction of the effect remained consistent, the magnitude was somewhat attenuated, indicating that the presence of publication bias could have a moderate impact on the strength of the evidence presented.

The overall certainty of evidence, as assessed using the GRADE framework, was rated as very low across all three postoperative timepoints. This evaluation was based on consistent downgrading across three domains: serious risk of bias due to limitations in study design and execution; serious inconsistency, reflected in substantial heterogeneity among trials; and serious imprecision, owing to small sample sizes and wide confidence intervals in several comparisons. While the pooled results indicated clinically meaningful reductions in pain that exceeded the MCID, these methodological concerns temper the strength of the conclusions. As such, the current evidence should be interpreted cautiously, and future trials employing rigorous design and standardized outcome reporting are needed to increase confidence in the findings.

This review underscores the importance of immediate postoperative pain management and suggests the need for a critical analysis of the benefits of various interventions in the days following surgery. The decreasing difference in pain perception between treatment and control groups by the seventh day indicates a natural progression of healing and recovery, potentially diminishing the relative impact of interventions over time.

Limitations of this review include the diversity of control groups and interventions, variability in study designs, and differences in sample sizes. Future research should focus on standardizing control groups, assessment periods, and outcome measurement methods to minimize variability and maximize data comparability. While no serious complications were reported in the included studies, most trials did not evaluate adverse events systematically. As such, the safety profile of these interventions remains insufficiently characterized.

## Conclusion

Leukocyte-Platelet Rich Fibrin (L-PRF) and 0.2% hyaluronic acid (HA) gel demonstrated the most excellent effectiveness in reducing postoperative pain following free gingival graft procedures, particularly within the first postoperative week, as measured by the Visual Analog Scale (VAS). However, methodological concerns, particularly regarding blinding and allocation concealment, along with high heterogeneity among studies, limit the generalizability of these findings. Standardizing control groups, assessment methods, and follow-up periods in clinical trials is crucial to strengthening future evidence. Evaluating the cost-effectiveness of L-PRF in resource-limited settings is also essential. Additionally, palatal protection plates showed promising results in pain reduction and wound protection, providing a practical and accessible alternative for managing postoperative discomfort. Despite these limitations, the findings support the clinical use of L-PRF and HA gel, along with palatal protection plates, for postoperative pain management, offering practical benefits for periodontology and oral surgery.

## References

[1] Zucchelli G, Mele M, Stefanini M, Mazzotti C, Marzadori M, Montebugnoli L (2010). Patient morbidity and root coverage outcome after subepithelial connective tissue and de‐epithelialized grafts: a comparative randomized‐controlled clinical trial. Journal of Clinical Periodontology.

[2] Langer B, Calagna L (1980). The subepithelial connective tissue graft. The Journal of Prosthetic Dentistry.

[3] Edel A (1974). Clinical evaluation of free connective tissue grafts used to increase the width of keratinised gingiva. Journal of Clinical Periodontology.

[4] Hürzeler MB, Weng D (1999). A single-incision technique to harvest subepithelial connective tissue grafts from the palate. The International journal of periodontics & restorative dentistry.

[5] Raetzke PB (1985). Covering Localized Areas of Root Exposure Employing the “Envelope” Technique. Journal of Periodontology.

[6] Silva ALM, de Souza JAC, Nogueira TE (2023). Postoperative local interventions for the palate as a gingival graft donor area: a scoping review. Clinical oral investigations.

[7] Eldien AMS, Hassabou NF (2022). Clinical and cytological assessment of platelet-rich fibrin versus topical ozonated oil in palatal wound healing after free gingival graft harvesting: Randomized controlled trial. Journal of Oral and Maxillofacial Surgery, Medicine, and Pathology.

[8] Morshedzadeh G, Aslroosta H, Vafaei M (2022). Effect of GaAlAs 940 nm Photobiomodulation on palatal wound healing after free gingival graft surgery: a split mouth randomized controlled clinical trial. BMC oral health.

[9] Ozcan M, Ucak O, Alkaya B, Keceli S, Seydaoglu G, Haytac MC (2017). Effects of Platelet-Rich Fibrin on Palatal Wound Healing After Free Gingival Graft Harvesting: A Comparative Randomized Controlled Clinical Trial. The International journal of periodontics & restorative dentistry.

[10] Samani MK, Saberi BV, Ali Tabatabaei SM, Moghadam MG (2017). The clinical evaluation of platelet-rich plasma on free gingival graft's donor site wound healing. European Journal of Dentistry.

[11] Shanmugam M, Arun R, Karthik S, Kumar TSS, Arun KV (2010). Clinical and histological evaluation of two dressing materials in the healing of palatal wounds. Journal of Indian Society of Periodontology.

[12] Ustaoğlu G, Ercan E, Tunali M (2016). The role of titanium-prepared platelet-rich fibrin in palatal mucosal wound healing and histoconduction. Acta odontologica Scandinavica.

[13] Madi M, Kassem A (2017). Topical simvastatin gel as a novel therapeutic modality for palatal donor site wound healing following free gingival graft procedure. Acta odontologica Scandinavica.

[14] Castro-Gaspar C, Olmedo-Gaya MV, Romero-Olid MN, Lisbona-Gonzalez MJ, Vallecillo-Rivas M, Reyes-Botella C (2021). Comparison between Tissue Adhesive Cyanoacrylate and Suture for Palatal Fibromucosa Healing: A Randomized Controlled Study. Materials.

[15] Alghriany AA, Ali AU, Khallaf ISA, Hassan AS, Sayed MA, Fikry AM (2024). Clinical effectiveness of orange peel polymethoxy-flavonoids rich fraction as a palatal dressing material compared to Alveogyl: randomized clinical trial. Scientific Reports.

[16] Heidari M, Paknejad M, Jamali R, Nokhbatolfoghahaei H, Fekrazad R, Moslemi N (2017). Effect of laser photobiomodulation on wound healing and postoperative pain following free gingival graft: A split-mouth triple-blind randomized controlled clinical trial. Journal of photochemistry and photobiology B, Biology.

[17] Patarapongsanti A, Bandhaya P, Sirinirund B, Khongkhunthian S, Khongkhunthian P (2019). Comparison of platelet-rich fibrin and cellulose in palatal wounds after graft harvesting. Journal of investigative and clinical dentistry.

[18] Karimi MR, Mansouri SS, Anoosh G, Montazeri M, Taheri N (2024). Effect of Cyanoacrylate Adhesive on Palatal Wound Healing Following Free Gingival Grafting: A Clinical Trial. Journal of Research in Dental and Maxillofacial Sciences.

[19] Eltas A, Dengizek Eltas Ş, Uslu MÖ, Ersöz M (2018). Evaluation of Patient Discomfort at the Palatal Donor Site Following Free Gingival Graft Procedures: A Randomized Controlled Clinical Trial. Journal of Periodontology & Implant Dentistry.

[20] Femminella B, Iaconi MC, Di Tullio M, Romano L, Sinjari B, D’Arcangelo C (2016). Clinical Comparison of Platelet‐Rich Fibrin and a Gelatin Sponge in the Management of Palatal Wounds After Epithelialized Free Gingival Graft Harvest: A Randomized Clinical Trial. Journal of Periodontology.

[21] Kızıltoprak M, Uslu MÖ (2020). Comparison of the effects of injectable platelet-rich fibrin and autologous fibrin glue applications on palatal wound healing: a randomized controlled clinical trial. Clinical Oral Investigations.

[22] Sharma V, Kumar A, Puri K, Bansal M, Khatri M (2019). Application of platelet-rich fibrin membrane and collagen dressing as palatal bandage for wound healing: A randomized clinical control trial. Indian journal of dental research : official publication of Indian Society for Dental Research.

[23] Soliman M, al barbary a, Elbattawy W, Shoeib M (2022). Clinical Comparison of Topical Application of Platelet Rich Fibrin Versus Application of Hyaluronic Acid Gel In Pain Management And Wound Healing After Free Gingival Graft Harvesting: A Randomized Controlled Clinical Trial. Egyptian Dental Journal.

[24] SqMH Scott, Lacy JA, Palaiologou AA, Kotsakis GA, Deas DE, Mealey BL (2024). Donor site wound healing following free gingival graft surgery using platelet rich fibrin: A randomized controlled trial. Journal of Periodontology.

[25] Tavelli L, Barootchi S, Nguyen TVN, Tattan M, Ravidà A, Wang HL (2018). Efficacy of tunnel technique in the treatment of localized and multiple gingival recessions: A systematic review and meta-analysis. J Periodontol.

[26] Bitencourt FV, Cardoso De David S, JdS Schutz, Otto Kirst A, Visioli F, Fiorini T (2022). Minimizing patient morbidity after free gingival graft harvesting:A triple-blind randomized-controlled clinical trial. Clinical oral implants research.

[27] Miguel MMV, Mathias-Santamaria IF, Rossato A, Ferraz LFF, Figueiredo-Neto AM, de Marco AC (2021). Microcurrent electrotherapy improves palatal wound healing: Randomized clinical trial. Journal of periodontology.

[28] Miguel MMV, Mathias-Santamaria IF, Rossato A, Ferraz LFF, Rangel TP, Casarin RCV (2021). Enamel matrix derivative effects on palatal mucosa wound healing: Randomized clinical trial. Journal of periodontal research.

[29] Soheilifar S, Bidgoli M, Hooshyarfard A, Shahbazi A, Vahdatinia F, Khoshkhooie F (2018). Effect of Oral Bromelain on Wound Healing, Pain, and Bleeding at Donor Site Following Free Gingival Grafting: A Clinical Trial. Journal of dentistry.

[30] Kadkhoda Z, Tavakoli A, Chokami Rafiei S, Zolfaghari F, Akbari S (2020). Effect of Amniotic Membrane Dressing on Pain and Healing of Palatal Donor Site: A Randomized Controlled Trial. International journal of organ transplantation medicine.

[31] Isler SÇ, Uraz A, ŞEngÜL J, ÇAkiroĞLu M, Bakirarar B, ÇEtİNer D (2019). Evalution Of the Patiens Oral Health Related Quality of Life After Harvesting Free Gingival Graft. Cumhuriyet Dental Journal.

[32] Taşdemir Z, Alkan BA, Albayrak H (2016). Effects of Ozone Therapy on the Early Healing Period of Deepithelialized Gingival Grafts: A Randomized Placebo-Controlled Clinical Trial. Journal of periodontology.

[33] Alpan AL, Cin GT (2023). Comparison of hyaluronic acid, hypochlorous acid, and flurbiprofen on postoperative morbidity in palatal donor area: a randomized controlled clinical trial. Clinical oral investigations.

[34] Isler SC, Uraz A, Guler B, Ozdemir Y, Cula S, Cetiner D (2018). Effects of Laser Photobiomodulation and Ozone Therapy on Palatal Epithelial Wound Healing and Patient Morbidity. Photomedicine and laser surgery.

[35] Keceli HG, Aylikci BU, Koseoglu S, Dolgun A (2015). Evaluation of palatal donor site haemostasis and wound healing after free gingival graft surgery. Journal of clinical periodontology.

[36] Vieira JPPG, Demarco AC, Braulino A, Filho M, Aparecida M (2010). Clinical Study of Laser Biomodulation.

[37] Gatti F, Iorio-Siciliano V, Scaramuzza E, Tallarico M, Vaia E, Ramaglia L (2023). Patient-reported outcome measures of leucocyte- and platelet-rich fibrin (L-PRF) or hemostatic agent application at palatal donor sites after free gingival graft harvesting: a randomized controlled clinical trial. Quintessence international.

[38] Yilmaz M, Kayaalti-YÜKsek S, Karaduman B (2022). The Effects of Cyanoacrylate on Clinical Healing and Self-Reported Outcomes Following Free Gingival Graft Surgery: A Randomized Clinical Study. Clinical and Experimental Health Sciences.

[39] Cairo F, Barootchi S, Tavelli L, Barbato L, Wang HL, Rasperini G (2020). Aesthetic‐And patient‐related outcomes following root coverage procedures: A systematic review and network meta‐analysis. Journal of Clinical Periodontology.

[40] Hassan A, Akl N, Adel-Khattab D (2020). Platelet Rich Fibrin Versus Hyaluronic Acid as palatal wound Dressings following epithelialized free gingival graft harvest: A randomized controlled clinical trial. Egyptian Dental Journal.

[41] Ehab K, Abouldahab O, Hassan A, Fawzy El-Sayed KM (2020). Alvogyl and absorbable gelatin sponge as palatal wound dressings following epithelialized free gingival graft harvest: a randomized clinical trial. Clinical oral investigations.

[42] Alkaya B, Kayhan HG, Temmerman A, Haytac MC, Teughels W (2023). Pre-operative, chair-side Zn-containing surgical stents affect morbidity and wound healing after free gingival graft harvesting: a randomized clinical trial. Clinical Oral Investigations.

[43] Hassan A, Ahmed E, Ghalwash D, Elarab AE (2021). Clinical Comparison of MEBO and Hyaluronic Acid Gel in the Management of Pain after Free Gingival Graft Harvesting: A Randomized Clinical Trial. International Journal of Dentistry.

[44] Yıldırım S, Özener HÖ, Doğan B, Kuru B (2018). Effect of topically applied hyaluronic acid on pain and palatal epithelial wound healing: An examiner-masked, randomized, controlled clinical trial. Journal of periodontology.

[45] Yussif N, Wagih R, Selim K (2021). Propylene mesh versus acrylic resin stent for palatal wound protection following free gingival graft harvesting: a short-term pilot randomized clinical trial. BMC oral health.

[46] Ustaoglu G, Ercan E, Tunali M (2017). Low-Level Laser Therapy in Enhancing Wound Healing and Preserving Tissue Thickness at Free Gingival Graft Donor Sites: A Randomized, Controlled Clinical Study. Photomedicine and laser surgery.

[47] Pekbağrıyanık T, Dadas FK, Enhoş Ş (2021). Effects of non-thermal atmospheric pressure plasma on palatal wound healing of free gingival grafts: a randomized controlled clinical trial. Clinical oral investigations.

[48] Mutallibli A, Saǧlam M (2024). Comparison of the effect of A-PRF and L-PRF application to palatal donor sites on quality of life and wound healing after free gingival graft surgery. Quintessence International.

[49] Basma HS, Saleh MHA, Abou-Arraj RV, Imbrogno M, Ravida A, Wang H-L (2023). Patient-reported outcomes of palatal donor site healing using four different wound dressing modalities following free epithelialized mucosal grafts: A four-arm randomized controlled clinical trial. Journal of periodontology.

[50] Meza-Mauricio J, Mourão ERST, Oliveira Marinho K, Vergara-Buenaventura A, Mendoza-Azpur G, Muniz FWMG (2023). Effect of collagen sponge and flowable resin composite on pain management after free gingival graft harvesting: A randomized controlled clinical trial. European journal of oral sciences.

[51] Sankar AR, Gujjari SK, P K K (2021). To Evaluate Aloe Vera- Chitosan Based Surgical Dressing to Chitosan Dressing Alone on the Healing of Palatal Donor Sites - A Pilot Study. International Journal of Research in Pharmaceutical Sciences.

[52] Spin JR, de Oliveira GJPL, Spin-Neto R, Herculano RD, Marcantonio RAC (2021). Effect of natural latex membranes on wound repair of palate donor areas: A pilot randomized controlled trial study, including the membranes characterization. Materials Today Communications.

[53] Khalil S, Habashneh RA, Alomari S, Alzoubi M (2022). Local application of hyaluronic acid in conjunction with free gingival graft: a randomized clinical trial. Clinical Oral Investigations.

[54] Bahammam MA (2018). Effect of platelet-rich fibrin palatal bandage on pain scores and wound healing after free gingival graft: a randomized controlled clinical trial. Clinical oral investigations.

[55] Tavelli L, Asa'ad F, Acunzo R, Pagni G, Consonni D, Rasperini G (2018). Minimizing Patient Morbidity Following Palatal Gingival Harvesting: A Randomized Controlled Clinical Study. The International journal of periodontics & restorative dentistry.

[56] Tavelli L, Ravidà A, Saleh MHA, Maska B, Del Amo FS-L, Rasperini G (2019). Pain perception following epithelialized gingival graft harvesting: a randomized clinical trial. Clinical oral investigations.

[57] Taşdemir Z, Alkan BA, Albayrak H (2016). Effects of Ozone Therapy on the Early Healing Period of Deepithelialized Gingival Grafts: A Randomized Placebo‐Controlled Clinical Trial. Journal of Periodontology.

[58] Tavelli L, Ravidà A, Lin GH, Del Amo FS, Tattan M, Wang HL (2019). Comparison between Subepithelial Connective Tissue Graft and De-epithelialized Gingival Graft: A systematic review and a meta-analysis. J Int Acad Periodontol.

[59] Bertl K, Melchard M, Pandis N, Müller-Kern M, Stavropoulos A (2017). Soft tissue substitutes in non-root coverage procedures: a systematic review and meta-analysis. Clinical oral investigations.

[60] Delgado DA, Lambert BS, Boutris N, McCulloch PC, Robbins AB, Moreno MR (2018). Validation of Digital Visual Analog Scale Pain Scoring With a Traditional Paper-based Visual Analog Scale in Adults. Journal of the American Academy of Orthopaedic Surgeons Global research & reviews.

[61] Borie E OD, Orsi IA, Garlet K, Weber B, Beltrán V, Fuentes R (2015). Platelet-rich fibrin application in dentistry: a literature review. Int J Clin Exp Med.

